# Gene networks and expression quantitative trait loci associated with adjuvant chemotherapy response in high-grade serous ovarian cancer

**DOI:** 10.1186/s12885-020-06922-1

**Published:** 2020-05-13

**Authors:** Jihoon Choi, Danai G. Topouza, Anastasiya Tarnouskaya, Sean Nesdoly, Madhuri Koti, Qing Ling Duan

**Affiliations:** 1 Department of Biomedical and Molecular Sciences, Queen’s University, Kingston, Ontario Canada; 2 School of Computing, Queen’s University, Kingston, Ontario Canada

**Keywords:** Chemotherapy resistance, Co-expression network analysis, Valosin containing protein, Expression quantitative trait loci, The Cancer Genome Atlas, High-grade serous ovarian carcinoma, Genome-wide association study, Differential gene expression analysis, Transcriptomics, Genomics

## Abstract

**Background:**

A major impediment in the treatment of ovarian cancer is the relapse of chemotherapy-resistant tumors, which occurs in approximately 25% of patients. A better understanding of the biological mechanisms underlying chemotherapy resistance will improve treatment efficacy through genetic testing and novel therapies.

**Methods:**

Using data from high-grade serous ovarian carcinoma (HGSOC) patients in the Cancer Genome Atlas (TCGA), we classified those who remained progression-free for 12 months following platinum-taxane combination chemotherapy as “chemo-sensitive” (*N* = 160) and those who had recurrence within 6 months as “chemo-resistant” (*N* = 110). Univariate and multivariate analysis of expression microarray data were used to identify differentially expressed genes and co-expression gene networks associated with chemotherapy response. Moreover, we integrated genomics data to determine expression quantitative trait loci (eQTL).

**Results:**

Differential expression of the Valosin-containing protein (*VCP*) gene and five co-expression gene networks were significantly associated with chemotherapy response in HGSOC. *VCP* and the most significant co-expression network module contribute to protein processing in the endoplasmic reticulum, which has been implicated in chemotherapy response. Both univariate and multivariate analysis findings were successfully replicated in an independent ovarian cancer cohort. Furthermore, we identified 192 cis-eQTLs associated with the expression of network genes and 4 cis-eQTLs associated with *BRCA2* expression.

**Conclusion:**

This study implicates both known and novel genes as well as biological processes underlying response to platinum-taxane-based chemotherapy among HGSOC patients.

## Background

Ovarian cancer is the most lethal gynecological malignancy and the 8th leading cause of cancer death in women around the world [1]. According to the Global Cancer Observatory report in 2012, ovarian cancer accounts for 3.6% of all cancer cases and 4.3% of all cancer related deaths worldwide [2]. High-grade serous ovarian carcinoma (HGSOC) is the most common form of ovarian cancer that accounts for up to 70% of all cases [3]. Routine diagnosis is often difficult due to the lack of mass screening methods and the heterogenous manifestations of symptoms, which results in approximately 75% of HGSOC patients diagnosed with advanced stages [4]. The average 5-year survival rates are 39% for Stage 3 and 17% for Stage 4 cancers [1].

The current standard of care for ovarian cancer is aggressive cytoreductive surgery followed by platinum-taxane combination chemotherapy [4]. However, this standard of care is not effective for all patients, with approximately 25% experiencing relapse within 6 months following chemotherapy containing platinum-based compounds, likely due to the development of antineoplastic resistance [5]. The median survival time for recurrent ovarian cancer ranges from 12 to 24 months [6, 7]. Treatment options for patients with recurrent ovarian cancer include non-platinum-based chemotherapy regimens, immunotherapy, and molecular targeted therapy [7, 8].

Ovarian cancer has a multifactorial etiology that includes genetic and non-genetic risk factors. An estimated 23% of cases are hereditary, but the majority are sporadic with multiple reported risk factors such as history of gravidity, infertility, and late age menopause [9, 10]. A better understanding of the etiology of ovarian cancer, as well as the genetic mechanisms underlying variable response to platinum-based chemotherapy, is needed for improved diagnosis and treatment. For example, previous studies reported that the *BRCA1* and *BRCA2* genes, which are associated with increased risk of ovarian cancer, harbor mutations associated with platinum drug sensitivity and survival [11]. Similarly, tumor suppressor genes such as *RB1*, *NF1*, *RAD51B*, *PTEN* have been associated with acquired chemotherapy resistance [12]. Earlier studies have also highlighted the importance of the immune system in the treatment of ovarian cancer. For example, loss of chemokines and disruptions to the IFN-γ pathway have been associated with poor treatment outcomes in HGSOC paients [13] whereas the NFκB signaling pathway and elevated expression of *STAT1* were associated with increased response to platinum therapy [14–16]. However, these known genetic variations do not account for all of the variability in chemotherapy response among HGSOC patients and there is currently no screening method to accurately predict prognosis prior to start of chemotherapy. Thus, further studies are necessary to determine additional modulators of chemotherapy response, which can be used as biomarkers for genetic testing.

The majority of earlier studies of chemotherapy response in ovarian cancer patients used univariate analysis of gene expression data known as differential gene expression (DGE) analysis. For example, DGE analysis identified genes correlated with ovarian cancer subtypes in the TCGA cohort [17], which have also been associated with differential response to platinum-based chemotherapy [18]. Moreover, similar univariate methods have been applied to investigate gene expression differences in cisplatin sensitive vs. resistant ovarian cancer cell lines after cisplatin exposure [19]. A limitation of DGE analysis is that it assumes each gene functions in isolation within the genome, which fails to capture the effects of complex gene-gene interactions. Our study of chemotherapy response in HGSOC patients applies a multivariate approach to identify groups of co-expressed genes, which may contribute to common biological pathways. These genes may each have modest effects that are not detected by conventional univariate analysis. Specifically, we applied Weighted Gene Co-expression Network Analysis [20] (WGCNA), which uses an unsupervised machine-learning algorithm to identify clusters of highly correlated or co-expressed genes. Moreover, we correlated sequence variations with co-expressed gene networks to identify expression Quantitative Trait Loci (eQTLs), which are potentially regulatory variants associated with gene expression. In addition, our study used gene expression data profiled from whole patient tumors, which were obtained during the initial cytoreductive surgery. This allows us to examine the tumor microenvironment and tumor cell intrinsic events which are difficult to study in cell-line derived expression datasets. A better understanding of the biological mechanisms regulating chemotherapy response will enable more effective treatment by improving the accuracy of genetic testing and identifying novel therapies for HGSOC patients.

## Methods

### Patient classification

We retrieved 587 high-grade serous ovarian carcinoma (HGSOC) patients with available clinical data from the Cancer Genome Atlas (TCGA) Genomic Data Commons (GDC) portal [21] using the TCGAbiolinks R/Bioconductor package [22]. We selected for patients who received platinum-based adjuvant chemotherapy, the majority of which (96%) also received taxane treatment (see Supplemental Table 1 for characteristics of the cohort). A small percentage of the cohort has received additional adjuvant therapies in combination with platinum compounds, such as gemcitabine (9%), doxorubicin (2.6%), topotecan (2.6%), bevacizumab (2.2%), and tamoxifen (2.2%) (Supplemental Table 2). The interval between a patient’s last primary chemotherapy treatment and the onset of a recurrent tumor or progression of an existing tumor was used as a metric for determining chemotherapy sensitivity. Patients who developed a new tumor in less than 6 months following their last primary chemotherapy treatment were defined as resistant (*N* = 110). In contrast, those who did not have a recurrent tumor event for over a year after their last primary chemotherapy treatment were defined as sensitive (*N* = 160). Individuals who had a recurrent tumor event between 6 months to 1 year following chemotherapy were excluded from the study. This strategy for dichotomizing resistant and sensitive patients was used to enrich for genetic differences.

### Transcriptomics data processing and analysis

#### Expression microarrays

Of the 270 HGSOC subjects classified as sensitive or resistant to chemotherapy, 238 (138 sensitive, 100 resistant) had primary tumor microarray expression data available (Affymetrix ht_hg_u133a chip) in the GDC portal. The robust multi-array average (RMA) method [23] in the *affy* package from Bioconductor [24] was used for background correction, log-transformation, and quantile normalization of the probe intensities. Two potential outliers and two duplicated samples were removed from the study during the quality control step using the *arrayQualityMetrics* [25] package (see Supplemental Data 1 for steps of pre-processing), resulting in 135 sensitive and 99 resistant HGSOC subjects in the expression set. Next, probes were filtered using the median absolute deviation (MAD) whereby the top 50% with highest variation (*n* = 11,107) were selected for analysis. This non-specific filtering step removed probes with low variability in expression across the cohort, which are not likely to be differentially expressed between sensitive and resistant patients, reducing the number of multiple testing corrections and, therefore, the likelihood of false positives.

#### Covariates

We assessed multiple potential confounders for correlation with therapeutic outcome including age, race, surgery (cytoreductive) outcome, cancer grade, and cancer stage (Supplemental Table 1). With the exception of age (*p* = 0.0041), all factors showed no significant difference between chemo-sensitive and chemo-resistant patients. For this reason, age at diagnosis was included as a covariate in all subsequent analyses.

#### Differential gene expression analysis

The *Limma* [26] package in Bioconductor [27] was used to identify differentially expressed genes between chemo-sensitive and resistant groups using linear models. The false discovery rate (FDR) method was employed as a measure for multiple testing correction to control for type I error.

#### Weighed gene co-expression network analysis (WGCNA)

We performed hierarchical clustering of genes using the R package *WGCNA* [20], which groups genes based on their similarity in expression. This was achieved by first creating a similarity matrix using Pearson correlation of expression among all genes. The resulting matrix was raised to a power of 9, as suggested by the soft-thresholding power estimation plot (Supplemental Fig. 1). Raising the correlation matrix to a power enriches for differences between weak and strong signals, allowing for better quantification of gene-gene interactions. The similarity matrix was transformed to a Topological Overlap Matrix (TOM), where the strength of association between a pair of genes is reinforced by the common neighbors shared by them. To avoid excessive splitting of genes into smaller modules, minimum module size was set to 30, split sensitivity (deep split) was set to 4, and modules with similar expression profiles were merged at a height of 0.5 (Supplemental Fig. 2). Using principal component analysis, we calculated the module eigengene for each co-expression cluster to summarize module gene expression with a single measure. Each module eigengene was tested for association with chemotherapy response using generalized linear models. Finally, we used *Cytoscape* [28], an open source bioinformatics platform, to visualize significant gene co-expression networks.

#### Gene function and pathway annotations

The Database for Annotation, Visualization and Integrated Discovery (DAVID) [29] was employed to identify biological pathways and functions that were enriched in each significant gene co-expression module. We also screened significant genes in the GeneMANIA [30] database to identify functional connections reported in published literature. Next, we searched the UCSC transcription factor binding site (TFBS) conservation sites track using DAVID to identify enriched motifs of transcription factors that may co-regulate genes within each cluster. Finally, we used the Drug–Gene Interaction database (DGIdb) [31], a public database with curation of data describing relationships between genes, chemicals, drugs, and pathological phenotypes, to identify genes with prior reported associations with chemotherapeutic agents.

#### Validation of differentially expressed gene

The Kaplan–Meier plotter tool was used to cross-validate the differential expression of *VCP* in an independent ovarian cancer cohort (GEO accession identifier: GSE9891) [32]. This replication cohort included gene expression profiling (Affymetrix Human Genome U133 Plus 2.0 Array) of 285 ovarian tumor samples. Patients were filtered to include those with cancer histology of serous carcinoma and who received chemotherapy containing a platinum compound to allow close comparison with the TCGA ovarian cancer cohort. This step omitted a total of 60 subjects from analysis, which included 21 with endometrioid carcinoma cases and 43 who did not receive platinum therapy (4 overlapping subjects). Thus, 225 patients remained for replication analysis. Patient survival was evaluated using a Cox proportional hazards model and progression-free survival (PFS) was the primary outcome used in the replication analysis [33].

#### Validation of co-expression networks

For validation of co-expression networks, the SurvExpress database was used, which allows users to validate the combined effect of multiple gene expression measures with a target trait [34]. The same cohort and filtering steps were used for validation (GSE9891, *N* = 225). The survival curve was evaluated using a Cox proportional hazards model and PFS was the primary outcome used in the replication analysis.

### Genomics data processing and analysis

#### Genomics data

Single nucleotide polymorphisms (SNPs) data from germline tissues (DNA extracted from blood or solid non-tumor ovarian tissue) were obtained from the TCGA legacy database. The Affymetrix Genome-Wide Human SNP Array 6.0 was used to capture genetic variations, which detected 906,600 SNPs. Of the 270 subjects from TCGA classified as resistant or sensitive to chemotherapy, 266 (157 sensitive and 109 resistant) had genotype data available.

#### Imputation

The imputation of autosomal chromosomes was performed using the Michigan imputation server pipeline [35]. We used the 1000 Genome Project phase 3 sequencing data (version 5) [36] reference panel for the imputation of missing genotypes. We then used Eagle v.2.3 [37] for phasing of the genotypes to their respective chromosomes. For the imputation of variants on the X chromosome, SHAPEIT [38] was used for phasing in combination with the 1000 genomes project phase 3 (version 5) reference panel (Supplemental Data 2).

#### Quality control

##### Subject level

Two pairs of individuals had a relatedness coefficient (pi-hat) > 0.9, which were likely duplicated samples. One subject from each pair was randomly removed from the dataset. Next, inbreeding coefficients (F) were computed for each subject using PLINK [39]. A total of 18 subjects with high homozygosity (F > 0.05) or heterozygosity (F < -0.05) rates were excluded. Moreover, genetic sex was estimated based on heterozygosity rates (F) of the X chromosome, and four subjects who had undefined genetic sex (F > 0.2) were removed from the study.

#### SNP level

SNPs with minor allele frequency (MAF) less than 1% or with genotyping call rate less than 90% were removed. This step removed 38,430,595 SNPs with MAF < 0.01, resulting in 9,528,963 SNPs to be used for further analysis.

#### Genome-wide association study

After imputation and quality control, 240 subjects (*N* = 142 sensitive, 98 resistant) and a total of 9,528,963 SNPs (MAF > 0.1) remained available for analysis (Supplemental Data 3). Plink (v.1.90) was used to compute genome wide and *BRCA1/2* targeted association analysis using a logistic regression model. We pruned variants in strong (r^2^ > 0.8) linkage disequilibrium (LD) within the *BRCA1/2* loci to determine independent association signals. Manhattan plot was generated using the qqman package in R [40].

#### Variant annotations

Variant Effect Predictor (VEP) [41] was used to predict the functional consequence of the identified variants. Similarly, the database of Genome-Wide Repository of Associations Between SNPs and Phenotypes (GRASP) [42] and Clinvar [43] were used to identify variants with known phenotype associations.

### Expression quantitative trait loci (eQTL) analysis

Common SNPs (MAF > 0.01) were tested for association with gene expressions of *BRCA1*, *BRCA2*, and co-expression networks using the *matrixeQTL* R package [44]. The correlation of a genotype with nearby gene expression indicates potential regulatory function of the SNP on the corresponding gene. These regulatory SNPs are known as cis-expression Quantitative Trait Loci (cis-eQTL). Cis-eQTLs are defined as correlated SNPs found within 1 Mb from the gene transcriptional start site (TSS).

## Results

**Fig. 1:**
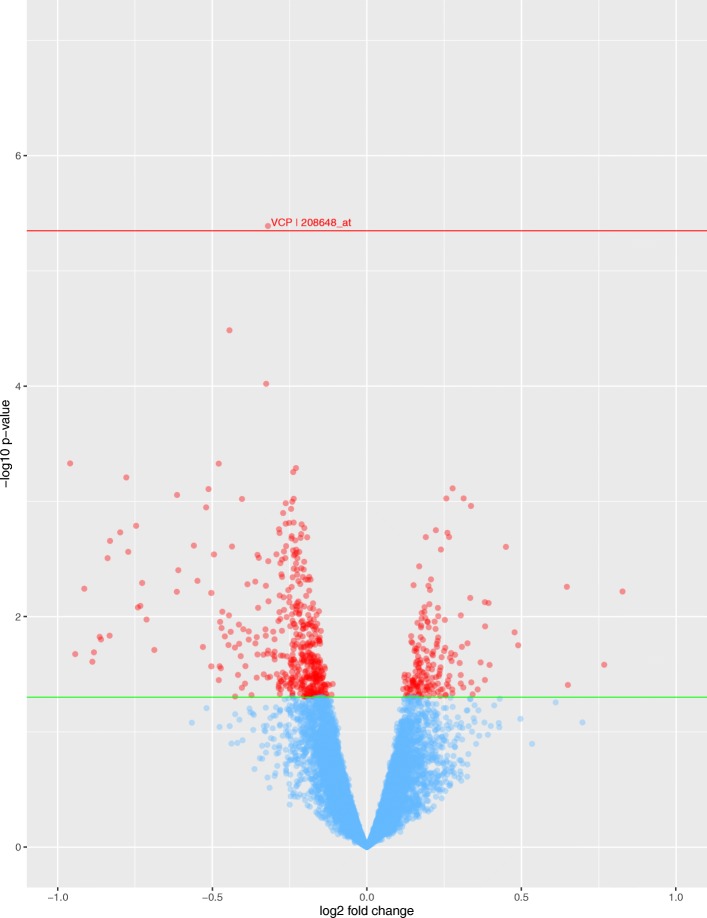
Differential expression analysis of platinum-taxane based chemotherapy response in HGSOC patients. Volcano plot showing univariate association analysis results. Red horizontal line demonstrates FDR-corrected *p* value (< 0.05) threshold. One probe, 208648_at, which maps to the Valosin-Containing Protein (*VCP*) gene is significantly differentially expressed and correlated with chemotherapy outcome after multiple testing correction. A total of 628 probes mapping to 534 unique genes are nominal associated, as indicated by the green line (*p* = 0.05). This figure was generated using the R package ggplot2 (v. 3.3.0)

**Fig. 2:**
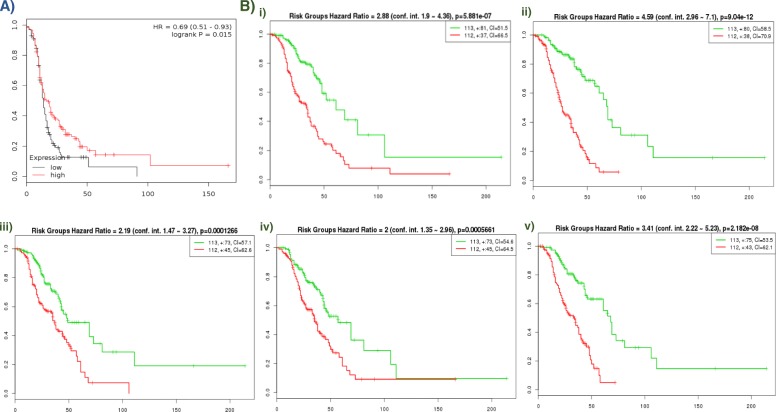
Kaplan-Meier (KM) plot of an independent ovarian cancer cohort for cross-validation of differentially expressed gene *VCP* and gene modules. KM plot shows the Progression-Free Survival (PFS) of the replication ovarian cancer cohort post platinum-based chemotherapy treatment. (A) shows the PFS of patients with high/low *VCP* expression, while (B) shows PFS of patients with high/low module-wide expression for (i) honeydew1, (ii) lightcyan1, (iii) lightpink3, (iv) orangered4, (v) skyblue3 modules. Red line in (A) indicates the PFS of patients with high *VCP* expression and black line indicates the PFS of patients with low *VCP* or module expression. Patients with high expression of *VCP* are associated with better PFS with statistical significance. Green line in (B) indicates the PFS of patients with high module-wide expression and red line indicates PFS of patients with low module-wide expression. Similarly, patients with high module wide expression are associated with better PFS with statistical significance. This figure was generated using the web-interface of the Kaplan-Meier Plotter and survExpress

Univariate or differential gene expression (DGE) analysis was used to test the association of 11,107 probes with chemotherapy response in HGSOC patients from TCGA. This identified that low expression of a probe (208648_at) mapping to the Valosin Containing Protein (*VCP*) gene was significantly associated with resistance to chemotherapy (FDR adjusted *p*-value < 0.05; Fig. 1). Replication analysis in an independent ovarian serous cancer cohort following treatment with platinum antineoplastic agents using the Kaplan–Meier survival curve plotter demonstrated that low expression of *VCP* was associated with poor progression-free survival (*p* = 0.015) and shorter median survival time (Fig. 2a). In addition to *VCP*, DGE analysis yielded 628 probes mapping to 534 unique genes that were nominally correlated with chemotherapy response (unadjusted *p*-value < 0.05). We report these findings in Supplemental Table 3.

**Fig. 3:**
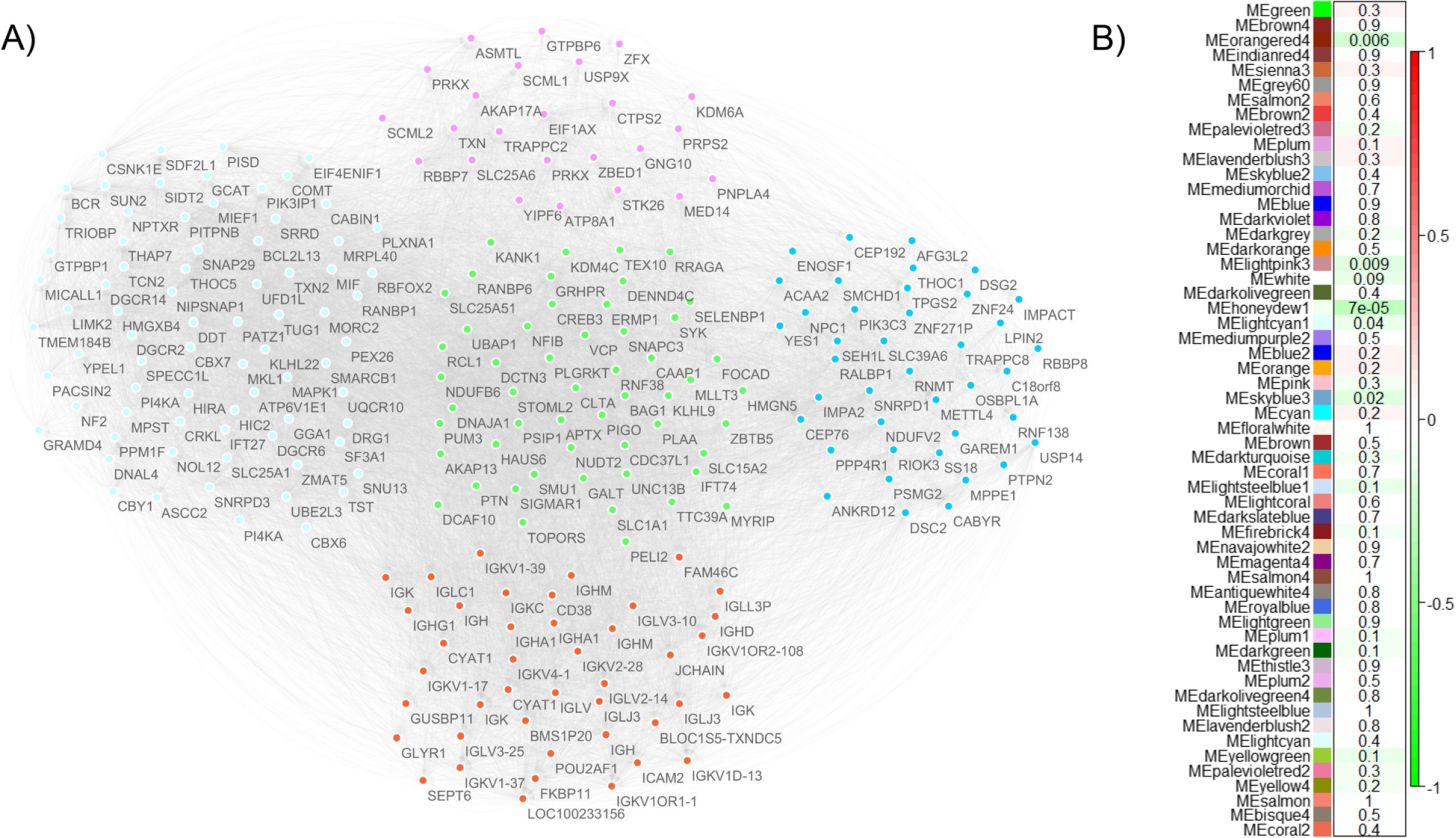
Gene co-expression modules correlated with platinum-based chemotherapy response. **a** Network plot displaying the five significant gene co-expression modules from *WGCNA*: honeydew1 - centre, lightcyan1 - left, lightpink3 - top, orangered4 - bottom, and skyblue3 - right. Nodes represent probes and edges are connections among the probes. Co-expressed probes (i.e. belonging to a single module) are indicated by the same color. **b** Heatmap demonstrating the association strength between the expression of gene modules and chemoresistance phenotype. Significance (*p*-value) of module-trait association is displayed beside each module. Colors represent strength of correlation, where red color indicates higher expression in chemoresistance population and green indicate higher expression in sensitive population. This figure was generated using Cytoscape (v.3.7.0)

The hierarchical clustering of genes using *WGCNA* resulted in 86 unique modules of co-expressed genes (Supplemental Table 4). Each module was assessed for association with chemotherapy response (results shown in Fig. 3b). Five gene clusters (*honeydew1, lightcyan1, lightpink3, orangered4, and skyblue3*) were significantly co-downregulated in platinum-resistant patients (*p* < 0.05) (Fig. 3a). These were validated in an independent ovarian cancer cohort by Tothill et al. [32] using SurvExpress, which demonstrated that the downregulation of the genes in the five modules was significantly associated with reduced patient survival (Fig. 2b). These five significant modules were annotated using DAVID, which identified gene enrichment for biological pathways including protein processing in the endoplasmic reticulum, apoptosis, negative regulation of the *Wnt* signaling pathway, transcription, immune response, and DNA double-strand break processing involved in repair via single-strand annealing. GeneMANIA analysis showed that genes in these modules were previously reported in 49 publications, some of which documented associations with oncogenic pathways and chemotherapeutic outcomes (Supplemental Data 4).

We performed a search of network module genes in the gene-drug interaction database (DGIdb) and found that 35 genes were associated with chemotherapeutic agents. These include: carboplatin and paclitaxel, which are often used as a first-line chemotherapy option for ovarian cancer patients; gemcitabine and bevacizumab, which are approved agents for the treatment of ovarian cancer; and various tyrosine kinase inhibitors (TKI), which are a type of targeted therapy commonly used for the treatment of chronic myeloid leukemia and other malignancies [45].

Furthermore, we identified common transcription factor binding sites located within genes from each module. For example, we identified that over 96% of genes (49/53 genes) found in the *honeydew1* module have a matching motif for the human organic cation transporter 1 transcription factor (OCT1). Similarly, we report that the acute myeloid leukemia 1 (AML1) motif maps to over 45% of genes found in *orangered4* module. Both of these transcription factors are associated with oncogenic processes and therapeutic outcome [46, 47]. A detailed list of functional annotations, transcription factors and pathways related to gene modules can be found in Supplemental Data 4.

Our GWAS of SNPs did not identify any variants correlated with chemotherapy response after multiple testing correction. The Manhattan plot (Supplemental Fig. 3) demonstrates that none of the SNPs meet the genome-wide significance threshold (*p* < 5 × 10–8), as indicated by the red horizontal line. This is likely due to insufficient statistical power resulting from the low number of subjects in the TCGA-OV cohort. Next, we performed a targeted association analysis of two well-known genes associated with ovarian cancer and chemotherapeutic outcomes: *BRCA1* and *BRCA2*. Of the 238 SNPs in *BRCA1* and 256 in *BRCA2*, we identified 56 independent variants in *BRCA1* and 86 such variants in *BRCA2* after pruning for LD (r^2^ > 0.8). Association analysis determined that 8 SNPs in *BRCA2* and 1 SNP in *BRCA1* were significantly associated with chemotherapy response. GRASP analysis identified that half of the identified *BRCA2* variants (rs11571686, rs7337574, rs10492397, rs1207952) have been previously associated with varied Low and High Density Lipoprotein (LDL/HDL) cholesterol levels. Similarly, annotation analysis using the Clinvar database reported that 4 of the associated variants in *BRCA2* (rs11571584, rs11571686, rs9567600, rs7337574) are linked with an increased risk of developing breast and ovarian cancer at an earlier age (Supplemental Table 5).

Next, SNPs were tested for correlation with the expression of the 5 network modules. This identified 192 cis-eQTLs associated with gene expression in co-expression networks. (Supplemental Data 5). Moreover, of the 8 significant SNPs found in *BRCA2*, 6 were identified as cis-eQTLs for nearby genes, including 4 that were specifically associated with *BRCA2* gene expression (Supplemental Table 5).

## Discussion

In this manuscript, we identified known and novel genes and gene networks correlated with variable response to platinum-taxane combination chemotherapy in HGSOC patients. Using a univariate analysis approach, we identified a differentially expressed gene encoding the valosin-containing protein (*VCP*) associated with sensitivity to chemotherapy. In addition, we applied a multivariate co-expression network analysis method which identified five clusters of co-expressed genes correlated with chemo-response. Genes in these modules were enriched for biological pathways such as protein processing in the endoplasmic reticulum, apoptosis, transcription, immune response, negative regulation of the *Wnt* signaling pathway and DNA double-strand break processing involved in repair via single-strand annealing. Moreover, we identified potentially regulatory variants (i.e. eQTLs) correlated with the expression of network genes and the tumor suppressor *BRCA2*, which has been previously associated with chemotherapy outcome in HGSOC. Our study contributes to a better understanding of the biological processes underlying chemotherapy response in HGSOC, which could help facilitate genetic testing and novel therapies.

The most significantly associated probe identified in the DGE analysis was for a gene encoding Valosin-containing protein (*VCP, p* = 3.91E-06). We have confirmed that this signal was replicated in an independent ovarian cancer cohort with statistical significance (*p* = 0.015). *VCP* plays a critical role in disintegrating large polypeptide cellular structures for further degradation by proteolytic enzymes. It functions to regulate important pathways of DNA repair, replication and cell cycle progression by removing faulty polypeptide structures from chromatin material, ribosomes, endoplasmic reticulum and mitochondria. *VCP* is an ovarian cancer-specific essential gene as demonstrated by a pooled short hairpin RNA (shRNA) screen in 25 ovarian cancer cell lines [48], and is also essential in cyclin E1 overexpressing cisplatin-resistant ovarian cancer cells [49]. In alignment with these findings, *VCP* has been investigated as a drug target for ovarian cancer therapy. For example, Bastola *et. al.* (2016) reported that *VCP* inhibitors induce cell death in ovarian cancer cell lines through the endoplasmic reticulum stress pathway [50]. In addition, this study reported an association between low *VCP* expression and poor response to platinum-based chemotherapy in multiple ovarian cancer cohorts. *VCP* has also been previously identified as a potential biomarker for predicting the success of platinum-based chemotherapy in lung cancer patients [51].

In our co-expression network analysis, the gene module “*honeydew1*” showed the most significant correlation with chemotherapy response (*p* = 6.53e-05). This association signal was validated with statistical significance (*p* = 5.88e-07) in an independent ovarian cancer replication cohort. This module includes two probes that map to *VCP*, a gene that was associated with chemotherapy response in our DGE analysis. Genes in this module were associated with positive regulation of mitochondrial membrane potential, protein ubiquitination, mitosis, alternative splicing, and apoptotic processes. Pathway analysis showed that this module is involved in protein processing in the endoplasmic reticulum. A prior study found that *VCP* plays a crucial role in ovarian cancer cell survival through extraction and degradation of unfolded proteins in endoplasmic reticulum, and noted that lower expression of *VCP* was associated with poor response to platinum-based chemotherapy [50]. In alignment with this finding, genes co-expressed in the *honeydew1* module were co-downregulated in chemo-resistant patients.

The *honeydew1* module is composed of 76 probes mapping to 53 unique genes, and of these, 45 genes are located in chromosome 9, demonstrating the importance of chromosome 9 in the regulation of chemo-resistance in ovarian cancer. These findings support previous studies, where genetic imbalance and alterations in chromosome 9 have been associated with progression of ovarian cancer and increased cisplatin resistance [52]. Analysis of overrepresented transcription factor binding sites demonstrated that genes in this module may be co-regulated by a common transcription factor known as organic cation transporter 1 (OCT1). We found that over 96% of genes in this module (49/53 genes) contain a nucleotide motif bound by OCT1. Prior studies have reported that silencing OCT1 impaired cisplatin-induced apoptosis in esophageal cancer cells, and that cisplatin-resistant cells were already expressing significantly reduced levels of OCT1 [46]. Taken together, these findings characterize a network of co-expressed genes that is associated with chemotherapy response in ovarian cancer. Genes within this module may be co-regulated by the OCT1 transcription factor, which may be used as a novel potential target for ovarian cancer therapies.

The other four co-expression modules, which were also replicated in an independent cohort, include genes known to be involved in oncogenic process and drug response outcomes. For example, the *orangered4* module, which was downregulated in resistant patients, consists of genes associated with regulation of the immune response. Genes in this module are associated with functional annotation terms including immunoglobulin receptor binding, antigen binding, B cell receptor signaling pathway, and phagocytosis. The repression of patient immune response is a well-known cancer survival mechanism, which has been shown to play a role in chemotherapy resistance in HGSOC [14, 15]. In addition, 10 of the 22 genes in this module are enriched for a common transcription factor binding site: acute myeloid leukemia 1 protein (AML1). This transcription factor is involved in the haematopoiesis process and immune functions such as thymic T-cell development. AML1 expression was found to be associated with cancer cell proliferation, migration and invasion in ovarian cancer [47]. In addition, we found that the *lightpink3* module is strongly associated with the transcription regulation process, which plays a pivotal role in cancer progression [53]. Finally, genes in the *lightcyan1* and *skyblue3* modules are target regions of well-known chemotherapeutic agents for ovarian cancer such as carboplatin, paclitaxel, bevacizumab and gemcitabine. Many of these genes are also target regions for various TKIs, which have been reported to enhance the efficacy of cisplatin treatment and progression free survival in ovarian cancer [54, 55]. For instance, our DGIdb search showed that the expression of the non-receptor tyrosine kinase *YES1* (YES Proto-Oncogene 1, Src Family Tyrosine Kinase) in the *skyblue3* module and the serine/threonine kinase *MAPK1* (Mitogen-activated protein kinase 1) in the *lightcyan1* module are inhibited when TKIs are introduced (Dasatinib, Ibrutinib, Rebastinib, Ulixertinib, etc.) (Supplemental Data 4).

Targeted analysis of *BRCA1* and *BRCA2* SNPs demonstrated that 6 out of 9 variants associated with chemotherapy response were also cis-acting eQTLs, correlated with the expression of *BRCA2* as well as neighboring genes *N4BP2L1*, *N4BP2L2*, *FRY*, and *STARD13* (nominal *p*-value < 0.05). Both *BRCA2* and *STARD13* are well known tumor-suppressors, and upregulation of *N4BP2L1* and *N4BP2L2* has been associated with positive prognosis in ovarian cancer cases [56]. The majority of cis-eQTLs in *BRCA2* were associated with the upregulation of *BRCA2* in chemotherapy resistant patients (Supplemental Table 5). The downregulation of *BRCA2* reduces the expression of the homologous recombination (HR) pathway-associated *RAD51* protein and suppresses DNA repair in ovarian cancer cells, sensitizing them to cisplatin [57]. In addition, *BRCA2* upregulation has been shown to promote HR DNA repair and radioresistance in pancreatic cancer cells [58]. This finding indicates that the potential regulation of *BRCA2* expression by the cis-eQTLs we identified may enhance the HR pathway function in resistant patients. However, functional experiments are needed to confirm this finding. Finally, our annotation results show that half of chemotherapy response-associated variants in *BRCA2* are linked with LDL/HDL cholesterol levels (Supplemental Table 5). Prior studies of lung and ovarian cancers consistently reported that cholesterol levels may affect the efficacy of platinum-based and platinum-taxane chemotherapy [59, 60]. Our findings indicate a new link between genetic variants in *BRCA2* and chemotherapy response through cholesterol level regulation.

One limitation of our study is that 96% of the HGSOC patients from TCGA received a combination of taxane and platinum-based chemotherapy. Thus, our results reflect response to the combinatorial therapy of platinum and taxane. Further studies are needed to test the association signals between the identified genes and platinum-specific or taxane-specific resistance. Despite the successful replication of our findings in an independent ovarian cancer cohort, another limitation is that our analysis and validation results are in silico-based. Further experimental studies are necessary to test the effects of down- or up-regulation of VCP and other gene networks on sensitivity to platinum-taxane chemotherapy.

## Conclusion

In this study, we identified genes and gene networks correlated with chemotherapy response in high-grade serous ovarian cancer patients, which implicate both known and novel biological mechanisms. Specifically, we identified that reduced expression of *VCP* is associated with resistance to chemotherapy. This gene is critical for removing unfolded proteins from the endoplasmic reticulum and has been known to be associated with cancer cell survival and response to platinum-based chemotherapy. In addition, we identified a group of genes associated with chemotherapy sensitivity that are co-expressed with *VCP* on chromosome 9. Genes from this module are involved in the protein processing in the endoplasmic reticulum pathway, which has been previously implicated in chemotherapy resistance and cancer cell survival. Finally, we report potentially cis-acting regulatory variants in the *BRCA2* gene that are associated with varied expression of *BRCA2*. In summary, our study contributes to a better understanding of the biological mechanisms underlying chemotherapy response in high-grade serous ovarian cancer. Our findings could help improve future patient screening and therapeutics for ovarian cancer through the identification of gene signatures that may predict chemotherapy response, as well as the potential discovery of novel drug targets.

## Supplementary information

**Additional file 1: Supplemental Data 1.** Microarray QC pipeline.

**Additional file 2: Supplemental Data 2.** Genotype imputation log.

**Additional file 3: Supplemental Data 3.** Genotype QC pipeline.

**Additional file 4: Supplemental Data 4.** Annotation of WGCNA modules.

**Additional file 5: Supplemental Data 5.** eQTLs associated with WGCNA genes.

**Additional file 6: Supplemental Figure 1.** Selection of soft-thresholding power for weighted gene coexpression network analysis (WGCNA). Scale independence plot on the left shows the change of scale free fit index (r2) per every increment of power. The mean connectivity plot on the right shows the change of average connectivity between genes for each power change. These two plots give guidance in choosing the optimal power in transforming the similarity matrix. Results from both plots indicate that at power 9, network reaches optimal scale free fit index. This figure was generated using the R package WGCNA (v.1.66).

**Additional file 7: Supplemental Figure 2.** Module dendrogram retrieved from hierarchical clustering of module eigengenes. The figure shows the dendrogram (tree diagram) of modules identified from co-expression clustering analysis of WGCNA pipeline. We merged modules showing high similarity to reduce excessive split of genes into many small sized clusters. Red horizontal line shows the threshold we used to merge modules with high similarity. This figure was generated using the R package WGCNA (v.1.66).

**Additional file 8: Supplemental Figure 3.** Manhattan plot of genome-wide SNP association study (GWAS). The figure shows the association between each individual SNP and status of chemoresistance. Each dot in Manhattan plot represents an individual SNP, x-axis displays the chromosomes which the variants are from and y-axis shows -log10 transformed *p*-value. Blue horizontal line shows genome-wide suggestive significance threshold (10e-5) and red horizontal line shows the genome-wide significance threshold (5e-8). This figure was generated using the R package qqman (v.0.1.4).

**Additional file 9: Supplemental Table 1.** Summary statistics of the study cohort.

**Additional file 10: Supplemental Table 2.** Adjuvant treatments given to patients in the study cohort.

**Additional file 11: Supplemental Table 3.** List of all differentially expressed genes.

**Additional file 12: Supplemental Table 4.** List of all WGCNA modules.

**Additional file 13: Supplemental Table 5.** BRCA targeted analysis.

## Data Availability

Transcriptomics, genomics, and clinical data used for the analysis of TCGA-OV cohort can be accessed/downloaded from the Genomic Data Commons (GDC) Data Portal (https://portal.gdc.cancer.gov/). Gene expression and clinical data of the replication cohort can be accessed/downloaded from Gene Expression Omnibus (GEO) database (https://www.ncbi.nlm.nih.gov/geo/query/acc.cgi?acc=GSE9899). An initial version of this manuscript has been deposited in a non-commercial preprint repository (bioRxiv accession number: 740696) [61].

## Abbreviations

DGE: Differential Gene Expression
eQTL: Expression Quantitative Trait Loci
FDR: False Discovery Rate
GDC: Genomic Data Commons
GEO: Gene Expression Omnibus
GWAS: Genome-Wide Association Study
HDL: High Density Lipoprotein
HGSOC: High-Grade Serous Ovarian Carcinoma
HR: Homologous Recombination
KM: Kaplan-Meier
LD: Linkage Disequilibrium
LDL: Low Density Lipoprotein
MAD: Median Absolute Deviation
PFS: Progression-Free Survival
RMA: Robust Multiarray Average
shRNA: pooled short hairpin RNA
SNP: Single Nucleotide Polymorphism
TCGA: The Cancer Genome Atlas
TFBS: Transcription Factor Binding Site
TKI: Tyrosine Kinase Inhibitor
TOM: Topological Overlap Matrix
TSS: Transcriptional Start Site
WGCNA: Weighted Gene Co-expression Network Analysis
