# Supplementary material for: Gene networks and expression quantitative trait loci associated with adjuvant chemotherapy response in high-grade serous ovarian cancer
Source: BMC Cancer. 2020 May 13;20:413. doi: 10.1186/s12885-020-06922-1 (PMC7218510; doi:10.1186/s12885-020-06922-1)
Supplement: Supplementary file 2 — Additional file 2: Supplemental Data 2. Genotype imputation log. [file 12885_2020_6922_MOESM2_ESM.pdf]

TCGA-OV genotype imputation log

Variant imputation

Michigan imputation server (https://imputationserver.sph.umich.edu) was used to impute for missing genotypes.

Autosomes (chr 1 – 22)

Chromosomes: 1 2 3 4 5 6 7 8 9 10 11 12 13 14 15 16 17 18 19 20 21 22
SNPs: 868263
Chunks: 153
Datatype: unphased
Reference Panel: phase3
Phasing: eagle

Statistics:
Alternative allele frequency > 0.5 sites: 235,699
Reference Overlap: 99.67%
Match: 860,761
Allele switch: 0
Strand flip: 0
Strand flip and allele switch: 0
A/T, C/G genotypes: 0
Filtered sites:
Filter flag set: 0
Invalid alleles: 448
Duplicated sites: 2
NonSNP sites: 0
Monomorphic sites: 1,088
Allele mismatch: 3,105
SNPs call rate < 90%: 0

X chromosome

Chromosomes: X
SNPs: 36764
Chunks: 8
Datatype: unphased
Reference Panel: phase3
Phasing: shapeit

Statistics:
Alternative allele frequency > 0.5 sites: 0
Reference Overlap: 99.05%
Match: 26,353
Allele switch: 7,769
Strand flip: 0
Strand flip and allele switch: 0
A/T, C/G genotypes: 1,383
Filtered sites:
Filter flag set: 0
Invalid alleles: 0
Duplicated sites: 0
NonSNP sites: 0
Monomorphic sites: 785
Allele mismatch: 133
SNPs call rate < 90%: 0

Total number of variants

	**Autosomes**	**X-chromosome**
**Before imputation**	868,263	36,764
**After imputation**	46,181,723	1,778,607
