# Supplementary material for: Gene networks and expression quantitative trait loci associated with adjuvant chemotherapy response in high-grade serous ovarian cancer
Source: BMC Cancer. 2020 May 13;20:413. doi: 10.1186/s12885-020-06922-1 (PMC7218510; doi:10.1186/s12885-020-06922-1)
Supplement: Supplementary file 6 — Additional file 6: Supplemental Figure 1. Selection of soft-thresholding power for weighted gene coexpression network analysis (WGCNA). Scale independence plot on the left shows the change of scale free fit index (r2) per every increment of power. The mean connectivity plot on the right shows the change of average connectivity between genes for each power change. These two plots give guidance in choosing the optimal power in transforming the similarity matrix. Results from both plots indicate that at power 9, network reaches optimal scale free fit index. This figure was generated using the R package WGCNA (v.1.66). [file 12885_2020_6922_MOESM6_ESM.pdf]

Supplemental Figure 1

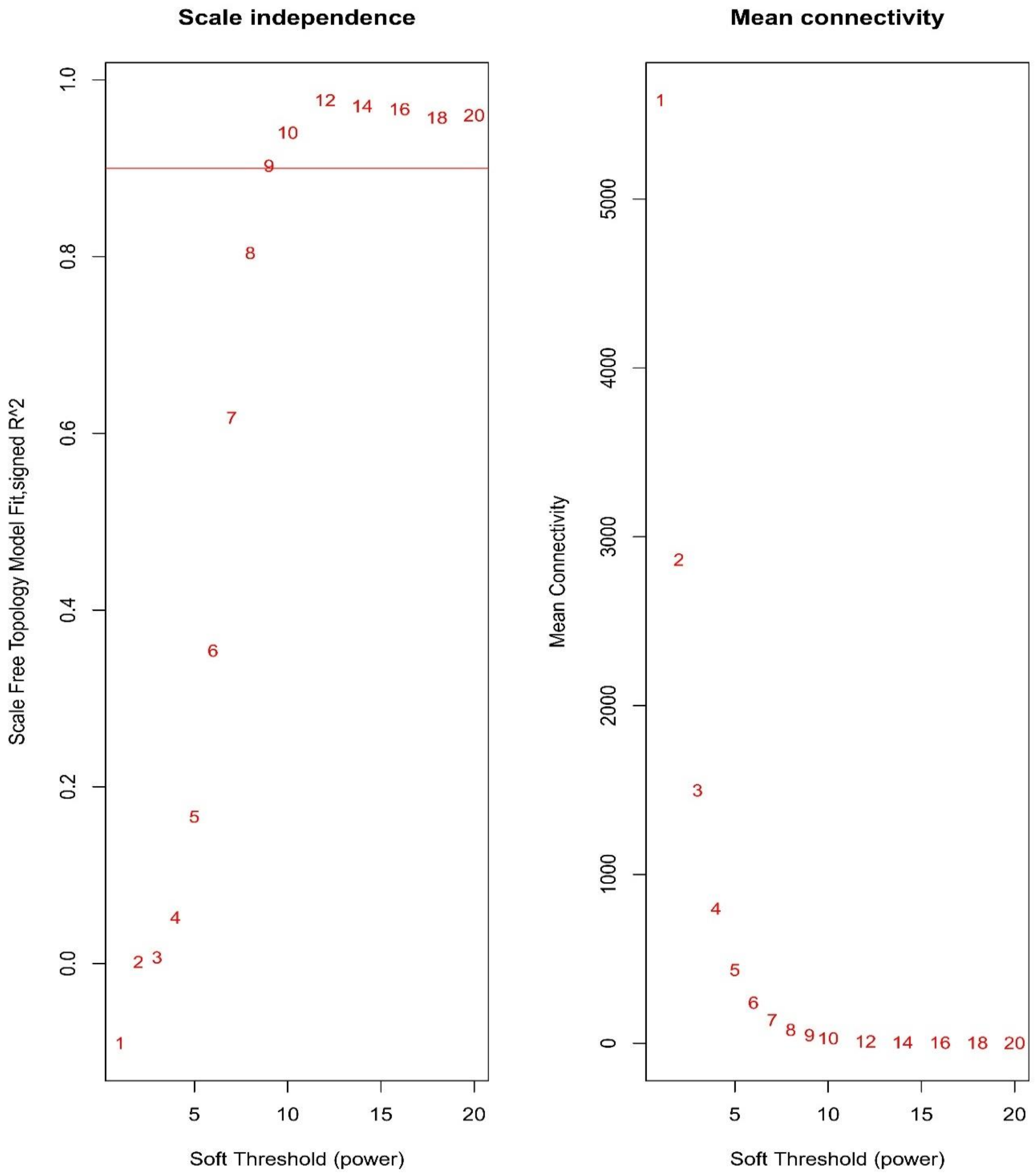

In this plot, we analyze the network topology at various soft thresholds (power). The assumption is that raising the similarity matrix by a power will enrich for differences between strong and weak signals. Scale independence plot (**left**) shows the change of scale-free fit index (r2) per change in power. The mean connectivity plot (**right**) shows the change of average connectivity between genes for each power increment. These results show that at power 9, scale free index reaches 0.9 (red horizontal line) and the network strongly resembles to a scale-free graph.
