# Supplementary material for: Gene networks and expression quantitative trait loci associated with adjuvant chemotherapy response in high-grade serous ovarian cancer
Source: BMC Cancer. 2020 May 13;20:413. doi: 10.1186/s12885-020-06922-1 (PMC7218510; doi:10.1186/s12885-020-06922-1)
Supplement: Supplementary file 7 — Additional file 7: Supplemental Figure 2. Module dendrogram retrieved from hierarchical clustering of module eigengenes. The figure shows the dendrogram (tree diagram) of modules identified from co-expression clustering analysis of WGCNA pipeline. We merged modules showing high similarity to reduce excessive split of genes into many small sized clusters. Red horizontal line shows the threshold we used to merge modules with high similarity. This figure was generated using the R package WGCNA (v.1.66). [file 12885_2020_6922_MOESM7_ESM.pdf]

Supplemental Figure 2

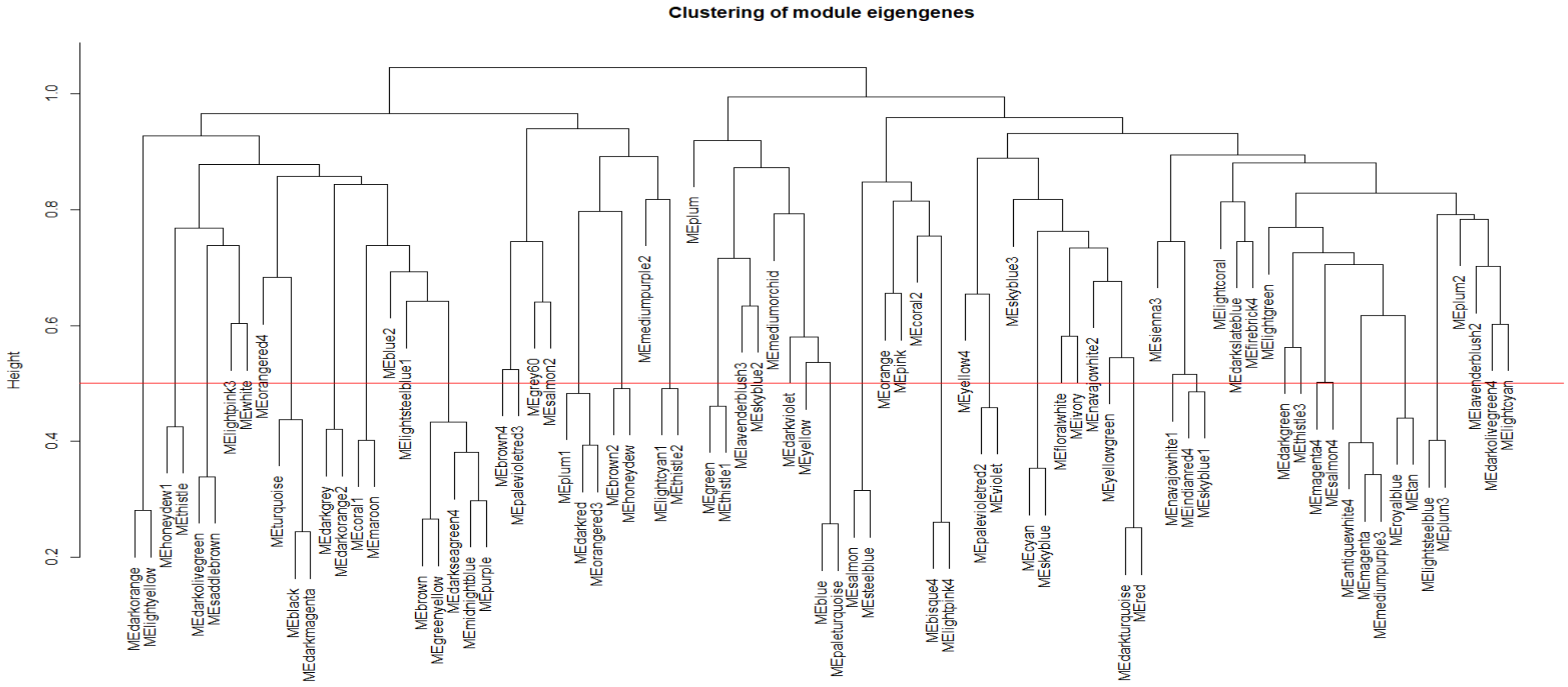

This plot shows every gene co-expression clusters identified from hierarchical clustering algorithm, where modules are represented by their eigengene. If similarity between two eigengenes are strongly correlated, we merge the two modules since genes in both modules would be strongly co-expressed. Red horizontal line in the plot indicates a height at which we merge modules.
