# Supplementary figures and images for: Gene networks and expression quantitative trait loci associated with adjuvant chemotherapy response in high-grade serous ovarian cancer
Source: BMC Cancer. 2020 May 13;20:413. doi: 10.1186/s12885-020-06922-1 (PMC7218510; doi:10.1186/s12885-020-06922-1)

Manhattan Plot

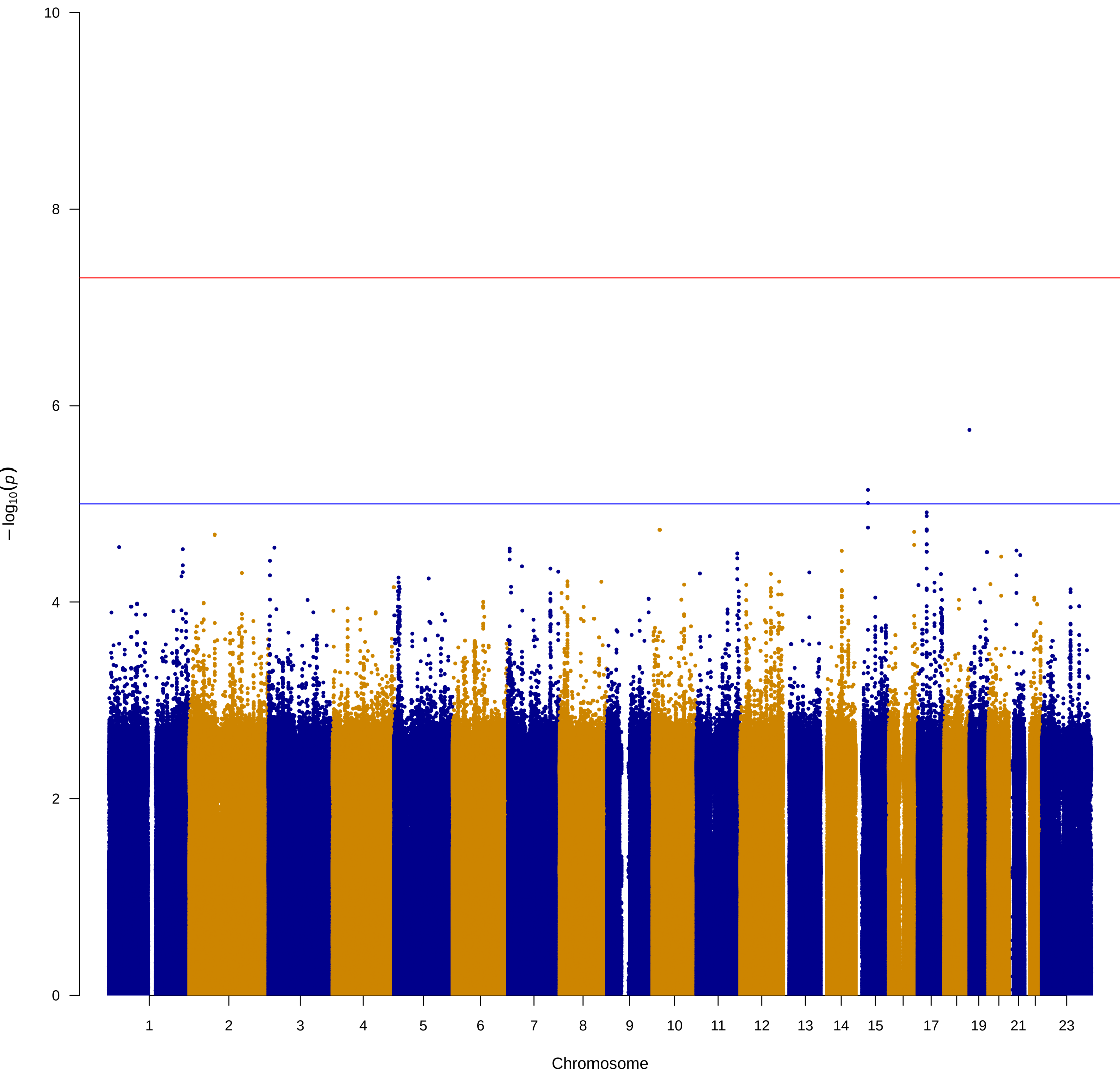

Supplement: Supplementary file 8 — Additional file 8: Supplemental Figure 3. Manhattan plot of genome-wide SNP association study (GWAS). The figure shows the association between each individual SNP and status of chemoresistance. Each dot in Manhattan plot represents an individual SNP, x-axis displays the chromosomes which the variants are from and y-axis shows -log10 transformed p-value. Blue horizontal line shows genome-wide suggestive significance threshold (10e-5) and red horizontal line shows the genome-wide significance threshold (5e-8). This figure was generated using the R package qqman (v.0.1.4). [file 12885_2020_6922_MOESM8_ESM.pdf]
